# Effect of Paper- and Computer-based Simulated Instructions on Clinical Reasoning Skills of Undergraduate Medical Students: A Randomized Control Trial

**DOI:** 10.7759/cureus.6071

**Published:** 2019-11-04

**Authors:** Masood Jawaid, Nighat Bakhtiar, Zubia Masood, Abdul-Khaliq Mehar

**Affiliations:** 1 Surgery, Darul Sehat Hospital, Karachi, PAK; 2 General Surgery, Dow University of Health Sciences, Karachi, PAK; 3 General Surgery, Ziauddin Medical University, Karachi, PAK

**Keywords:** clinical reasoning, computer based learning, simulation, blended learning

## Abstract

Background and objectives

Skilled clinical reasoning is a critical tool for physicians. Educators agree that this skill should be formally taught and assessed. Objectives related to the mastery of clinical reasoning skills appear in the documentation of most medical schools and licensing bodies. We conducted this study to assess the differences in clinical reasoning skills in medical students following paper- and computer-based simulated instructions.

Materials and methods

A total of 52 sixth semester medical students of the Dow University of Health Sciences were included in this study. A tutorial was delivered to all students on clinical reasoning and its importance in clinical practice. Students were divided randomly into two groups: group A received paper-based instructions while group B received computer-based instructions (as Flash-based scenarios developed with Articulate Storyline software [https://articulate.com/p/storyline-3]) focused on clinical reasoning skills in history-taking of acute and chronic upper abdominal pain. After one week, both groups were tested at two objective structured clinical examination (OSCE) stations to assess acute and chronic pain history-taking skills in relation to clinical reasoning.

Results

There were 27 students in group A and 25 students in group B. The mean OSCE score for group A (paper-based) was 28.6 ± 9.4 and that for group B (computer-based) was 38.5 ± 6.0. Group B’s mean score was statistically significantly greater (p < 0.001) than group A’s mean score for clinical reasoning skills.

Conclusion

A computer simulation program can enhance clinical reasoning skills. This technology could be used to acquaint students with real-life experiences and identify potential areas for more training before facing real patients.

## Introduction

Clinical reasoning is a process by which clinicians “collect cues, process the information, come to an understanding of a patient problem or situation, plan and implement interventions, evaluate outcomes, and reflect on and learn from the process” [1-2]. Clinical reasoning is an important element of essential medical practice. A positive impact occurs on patient outcomes when a doctor has valuable clinical reasoning ability. Physicians with poor clinical reasoning skills usually fail to achieve positive outcomes and, consequently, compromise patient safety.

The development of an essential level of clinical reasoning skills is not facilitated by current medical curricula [3]. Cognitive failure is at the root of 57% of poor clinical results according to Wilson et al [4]. Objectives related to the mastery of clinical reasoning skills appear in the documentation of most medical schools and licensing bodies, but they are not usually practiced properly.

To develop the skill set for the proper diagnosis and management of a deteriorating patient and for essential communication in real health care, teaching and learning opportunities must be started at the undergraduate level [5]. The dynamic nature of the current clinical practice situations needs medical graduates to acquire multifarious roles that require clinical reasoning abilities during their undergraduate education.

All educators agree that this skill should be formally taught and assessed, but, unfortunately, there are some systemic hurdles faced by the teachers and clinicians in fulfilling this requirement. These hurdles are especially apparent in the progressively demanding and unpredictable character of the current health care environment and the lack of time for clinical educators and teachers to think through clinical problems with students [6]. One approach to overcoming these hurdles is the use of simulation-based clinical reasoning skills, which are becoming more available due to advancements in technology [7]. Many studies have explored the use of simulation in the education of health care professionals, but thorough analyses remain difficult. Most studies were not focused on clinical reasoning skill development at the undergraduate level [8]. Data on clinical reasoning assessment through simulation pertain most often to nursing students [9]. Therefore, additional studies are required to determine and establish the usefulness of computer-based simulation as an educational tool to develop the clinical reasoning skills of undergraduate medical students.

We conducted this study to assess the differences in clinical reasoning skills taught through paper- and computer-based instructions (through simulation) among medical students.

## Materials and methods

This randomized controlled trial included undergraduate students at the Dow University of Health Sciences. This study was approved by the university's Institutional Review Board (number IRB-408/DUHS). A total of 52 sixth-semester medical students were included in this study. A clinical reasoning tutorial was delivered to all students by one of the authors (Masood Jawaid). Study participants were assigned into two groups using the Random Allocation Software, version 1.0 (http://mahmoodsaghaei.tripod.com/Softwares/randalloc.html). Students in group A (n = 27) received paper-based instructions from a recommended book for clinical practice of surgery [10]. Students in group B (n = 25) received computer-based instructions from Flash-based scenarios developed with Articulate Storyline software by Articulate Inc. (https://articulate.com/p/storyline-3), which focused on clinical reasoning skills in history-taking of acute and chronic upper abdominal pain. This development tool was used to construct screen content containing different skills, and the linkage between the screen content was used to reach the final diagnosis. After one week, both groups were tested by two objective structured clinical examination score (OSCE) stations on history-taking skills of acute and chronic pain in relation to clinical reasoning.

IBM SPSS Statistics for Windows, Version 20.0 (IBM Corp., Armonk, NY) was used to analyze data. The independent sample t-test was used to analyze the score difference between groups.

## Results

**Figure 1:**
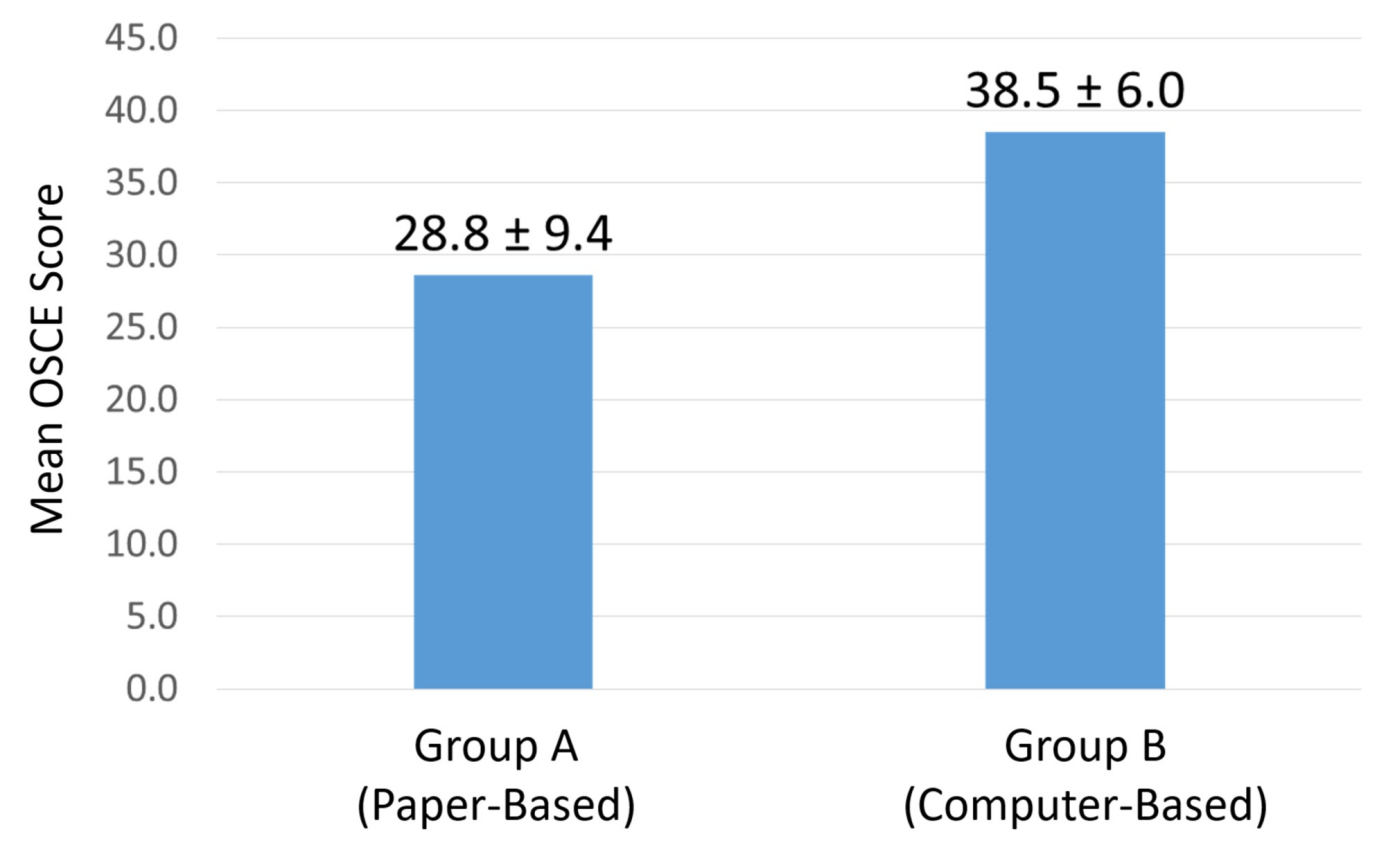
Mean OSCE Score OSCE: Objective Structured Clinical Examination.

The mean OSCE score of students in group A (paper-based instruction) was 28.6 ± 9.4. The mean OSCE score for students in group B (computer-based instruction) was 38.5 ± 6.0, which was statistically significantly higher than the mean group A score (p < 0.001) in clinical reasoning skills (Figure 1).

## Discussion

The significantly higher mean score achieved by students in the computer-based simulation program over the paper-based group indicates that computer-based instructions can enhance clinical reasoning skills of students. Our findings align with those of a study by Puri et al., whose results showed a mean post-test score of 6.65 ± 0.16 in the intervention group and 6.28 ± 0.29 in the control group (p < 0.05) [11]. Raidl et al. showed that computer-assisted simulations improved the clinical reasoning skills of dietetics students [12]. Similar results were also reported by Gilbart et al. in their study in which 92% of students indicated that simulation-based programming should be part of their clinical clerkship [13].

Most clinical faculties have very busy schedules that limit opportunities to properly develop clinical reasoning skills in their students. Adapting computer-based instruction and simulations can at least partially mitigate this systemic gap [6].

Our study was limited due to its relatively small number of participants. However, the degree to which sample size would have affected the results remains unclear. Another limitation was that the modules prepared did not cover multiple clinical scenarios and patients. We believe the students would have become more skilled otherwise. 

Additional research is needed to determine which type of simulation-based education is most appropriate for specific health professional student positions [14-15]. Blended learning courses that include both computer-based simulation instructions and traditional methods are highly useful for augmenting clinical learning in medical students. Medical educators should consider the blended learning system to enhance the clinical reasoning skills of their students [16-17].

## Conclusions

A computer simulation program can enhance clinical reasoning skills. This technology could be used to acquaint students with real-life experiences and identify potential areas for more training before facing real patients. On the other hand, it will help clinical educators using computer-based simulation to teach clinical reasoning and eventually the patients who are the receivers of care provided ultimately.
